# Modifications outside CDR1, 2 and 3 of the TCR variable β domain increase TCR expression and antigen-specific function

**DOI:** 10.3389/fimmu.2023.1148890

**Published:** 2023-04-12

**Authors:** Abdullah Degirmencay, Sharyn Thomas, Fiyaz Mohammed, Benjamin E. Willcox, Hans J. Stauss

**Affiliations:** ^1^ Institute of Immunity and Transplantation, Division of Infection and Immunity, University College London, London, United Kingdom; ^2^ Cancer Immunology and Immunotherapy Centre, Institute for Immunology and Immunotherapy, University of Birmingham, Edgbaston, Birmingham, United Kingdom

**Keywords:** TCR-T therapy, TCR (T cell receptor), TCRV, T cell function, framework engineering

## Abstract

T cell receptor (TCR) gene modified T cells are a promising form of adoptive cellular therapy against human malignancies and viral infections. Since the first human clinical trial was carried out in 2006, several strategies have been developed to improve the efficacy and safety of TCR engineered T cells by enhancing the surface expression of the introduced therapeutic TCRs whilst reducing the mis-pairing with endogenous TCR chains. In this study, we explored how modifications of framework residues in the TCR variable domains affect TCR expression and function. We used bioinformatic and protein structural analyses to identify candidate amino acid residues in the framework of the variable β domain predicted to drive high TCR surface expression. Changes of these residues in poorly expressed TCRs resulted in improved surface expression and boosted target cell specific killing by engineered T cells expressing the modified TCRs. Overall, these results indicate that small changes in the framework of the TCR variable domains can result in improved expression and functionality, while at the same time reducing the risk of toxicity associated with TCR mis-pairing.

## Introduction

The engineering of T cells with genes encoding TCR chains, or chimeric antigen receptors (CARs) is an efficient strategy to produce cells for antigen-specific T cell therapy in the clinical setting (1). TCR gene therapy typically relies on transferring antigen-specific T cell receptor alpha and beta chains into the autologous T cells obtained from patients (2). Several promising clinical benefits have been obtained using TCR gene therapy to target tumour associated antigens, cancer testis antigens and viral antigens (3–14). Nonetheless, certain drawbacks with this therapy have diminished its clinical efficacy and may pose a safety risk. For example, low expression levels of introduced TCRs may reduce T cell avidity and prevent recognition of target cells expressing low level of TCR-recognised target antigens (15). The expression levels of TCRs are determined by the amino acid composition of the variable alpha and variable beta domains, resulting in ‘dominant’ TCRs that are highly expressed on the surface of engineered T cells, and ‘weak’ TCRs that are poorly expressed (16).

A major safety concern relates to potential mis-pairing of endogenous and introduced TCR chains, which was shown to result in fatal autoimmunity in murine models of TCR gene therapy (17, 18), although such toxicities have not been observed in patients. Mis-pairing occurs between endogenous (end) α and introduced (int) β chains or vice versa during the pairing step of TCR chains in the endoplasmic reticulum (ER). Mis-pairing results in two additional receptor combinations (α_end_β_int_, α_int_β_end_) in transduced cells. In total, four different receptor combinations can result, αβ_end_, αβ_int_, α_end_β_int_, α_int_β_end_, only one of which is desired, namely αβ_int_.

Heemskerk et al. demonstrated that the quality of the endogenous TCR is a determining factor in the surface expression of introduced TCR, hence it is important that introduced TCRs are dominant over their endogenous competitor (19). To date, multiple strategies have been developed to improve the efficacy of TCR therapy and to tackle the issues of mis-pairing and suboptimal surface expression. These include TCR constant region murinisation (20, 21), introduction of an additional disulphide bond between Cα and Cβ (22, 23), codon optimisation (24), TCR domain swapping (25), single chain TCRs (26–29) and addition of accessory or co-stimulatory molecules (30, 31). Ablating the endogenous TCR using the zinc-finger (32), CRISPR (29, 33–35) or TALEN (36) technology has been employed to eliminate TCR mis-pairing and improve TCR expression levels.

TCR framework engineering is a technology that can improve TCR safety and efficacy, without the need for additional gene deletion, thus avoiding the safety concerns of the zinc-finger, CRISPR and TALEN technologies (16). Our previous framework engineering work has mostly focused on the TCR variable alpha domain (TRAV), without fully exploring the role of the TCR variable beta domain (TRBV). Here, we have analysed the framework amino acids of TRBV to further optimise TCR expression and antigen-specific function. We discovered that single amino acid changes in the TRBV framework region can enhance performance, and when combined with previously identified TRAV residue changes enable optimal TCR expression and function.

## Methods

### TCR gene usage

The weak1 TCR expressed the TRAV13-2/TRBV7-3 variable gene segments, the CMV1 TCR expressed TRAV24/TRBV6-5, the HA1.m2 TCR expressed TRAV13-1/TRBV7-9, and the HA1.m7 TCR expressed TRAV25/TRBV7-9.

### Cell culture

TCRαβ-deficit human Jurkat76 cells, HLA-A2^+^ T2 cells and human PBMCs were cultured in RPMI 1640 medium (Lonza) supplemented with 10% FCS, 1% L-Glutamine (Gibco, 2mM) and 1% Penicillin/Streptomycin (100U/ml). HEK293T (Human embryonic kidney epithelial) packaging cells were cultured in IMDM (Lonza) supplemented with 10% FCS, 1% L-Glutamine (Gibco, 2mM) and 1% Penicillin/Streptomycin (100U/ml).

### Primary human peripheral blood mononuclear cells

Human PBMCs were obtained from volunteer donors *via* the National Health Blood Transfusion Service (Approved by UCL Research Ethics Committee, Project ID/Title: 15887/001) and stored in the Biobank facility based at the Royal Free Hospital, London, UK, until use. 48h prior to retroviral transduction, bulk PBMCs were activated at 1x10^6^ cells/ml with 20μl anti-CD3/CD28 dyne beads (Gibco) and 30U/ml Roche IL-2.

### Retroviral vector and *In vitro* mutagenesis

Retroviral constructs were designed and produced as previously described (16). General structure of a TCR construct was consisting of a V5 sequence, a TCRα chain, a viral P2A sequence, two Myc sequence, a TCRβ chain, a viral T2A sequence, and truncated murineCD19.

*In vitro* mutagenesis was employed to implement the identified residue changes in TCR chains. Mutated primers were designed using the Agilent *in vitro* mutagenesis primer design tool. Quickchange II XL Site-Directed Mutagenesis Kit (Agilent Technologies) was used to change the framework region amino acid residues by PCR as per protocol. Produced DNAs were sent for Sanger sequencing to verify the presence of intended amino acid changes.

### Retrovirus production and transduction of the cells

1.8-2.0 x 10^6^ HEK293T packaging cells were plated in 10-cm tissue culture dishes in 8ml complete IMDM media. On the following day, cells underwent a 100% media change with 5ml fresh complete IMDM media 30 minutes prior to the transfection. Transfection master mix A was prepared with 1.5μg pCl-ampho retroviral packaging vector and 2.6μg of TCR DNA with dH_2_O to a final volume of 50μl. Master mix B was composed of 150μl Opti-MEM media and 10μl FugeneHD (Promega. Master mixes A and B were mixed and incubated for 20 minutes at room temperature, and then added to the transfection plates by droplets. On Day1 post transfection, cells were 100% media changes and given 5ml fresh complete RPMI media. On Day2 post transfection, retroviral supernatants were harvested either used directly for a transduction of the target cells or stored in -80° C freezer. Non-TC treated, 750 ul Retronectin (Takara) overnight coated 24-well plate was used in the transduction of the pre-activated bulk hPBMCs. Following the collecting of the Retronectin, 24-well plate were blocked by 2% BSA-PBS (Sigma-Aldrich) for 30 mins. Following the incubation, wells were washed with PBS by 2x times. Then, 500ul viral supernatant and 5x10^5^ Jurkat76 or 1x106 bulk hPBMCs were added each well, and the transduction was done by centrifuge with 32°C, 2000rpm, 1h30 mins configurations. Following the transduction, supernatant in each well was discarded, and cells were supplied with 2ml complete RPMI while bulk hPBMCs received additional 10U/ml Roche IL-2. On Day-3/4 post transduction, cells were stained for Live/dead, anti-human CD3, anti-mouse CD19, anti-human CD8, anti-Myc, anti-V5. Data was collected by LSRFortessa (BD Biosciences) and the analysis was done by FlowJo software. While transduced Jurkat76 cells pre-gated on live, singlets and CD19+, transduced bulk hPBMCs pre-gated on live, singlets, CD19+, CD3+CD8+. V5/TCRα and Myc/TCRβ staining was used for determining TCR expression in both Jurkat76 and bulk hPBMCs.

### Antibodies and peptides

The following antibodies were used: anti-human CD3-FITC (Clone: HIT3A; BD), anti-mouse CD19-eFluor450 (Clone: 1D3; Invitrogen), mouse anti-c-Myc (Bio-Rad), rabbit polyclonal V5-APC (abcam), anti-mouse IgG1-PE (Invitrogen), anti-human CD3-PE-Cy7 (Clone: SK7; Biolegend), anti-human CD8-FITC (Clone: OKT8) and Live/Dead-eFluor780 (Invitrogen), anti-human IL-2-APC (Clone: MQ1-17H12, eBioscience) and anti-human IFN-γ-PE (Invitrogen). Peptides used were: pCMVpp65 (NLVPMVATV) for CMV1 TCR and the pHA1 (VLHDDLLEA) for HA-1.m2 and HA-1.m7 TCRs. The pHA2 (YIGEVLVSV) peptide was used as a control peptide in the functional assays.

### Killing assay

7-10 days post-transduced bulk hPBMCs were employed in killing assays. HLA-A2^+^ T2 cells were loaded with cognate peptide were labelled with 0.02uM CFSE whilst cells loaded with control peptide were labelled with 0.2uM CFSE. Following peptide loading for 2h, T2 cells were mixed at 1:1 ratio and 1x10^5^ transduced bulk-T cells were co-cultured with 1x10^5^ mixed T2 cells for 18 hours. Cells were stained with anti-human CD3 Ab and Live/Dead antibodies, and data acquisition was done by LSRFortessa and analysed by FlowJo software. Antigen specific killing of T cells was calculated as % Specific Killing = 100- [(Relevant/Irrelevant T2 cells with T cells)/(Relevant/Irrelevant T2 cells with no T cells)]*100.

### TCR structural modelling

The weak TCR that was most extensively tested in our study comprised of TRAV13-2 and TRBV7-3. A molecular model of the TRAV13-2/TRBV7-3 TCR complex was generated as described previously (16). Models of weak to strong TCRs incorporating the 11 variable domain framework residues were generated using the I-TASSER (Iterative Threading ASSEmbly Refinement) server (37). For all modelling studies with I-TASSER, the target sequences were initially threaded through the PDB library by the meta threading server, LOMETS2. Continuous fragments were excised from LOMETS2 alignments and structurally reassembled *via* replica-exchange Monte Carlo simulations. The simulation trajectories were then clustered and used as the initial state for second round I-TASSER assembly simulations. Finally, lowest energy structural models were selected and refined by fragment-guided molecular dynamic simulations to optimize polar interactions and omit steric clashes. Analysis of molecular interactions was carried using the CCP4 suite (38). Model visualization was performed with COOT (39) and structural figures were generated using PyMOL (The PyMOL Molecular Graphics System, Version 1.8 Schrödinger, LLC).

### Intracellular cytokine staining

3x10^5^ T2 cells were stimulated with either relevant or irrelevant peptide for 2 hours. Then they were washed and re-suspended in RPMI and co-cultured with 7-10-day post-transduced 3x10^5^ bulk-T cells for 18 hours. Cells were stained with surface markers and washed. Then they were fixed by BD Cytofix/Cytoperm Kit and incubated for 20 minutes at 4°C. Afterwards they were washed and stained for IL-2 and IFN-γ and incubated for 1 hour at 4°C. Data acquisition was done by LSRFortessa and analysed by FlowJo software.

## Results

### A single amino acid change in the framework of TRBV improves TCR expression in human Jurkat76 cells

**Figure 1:**
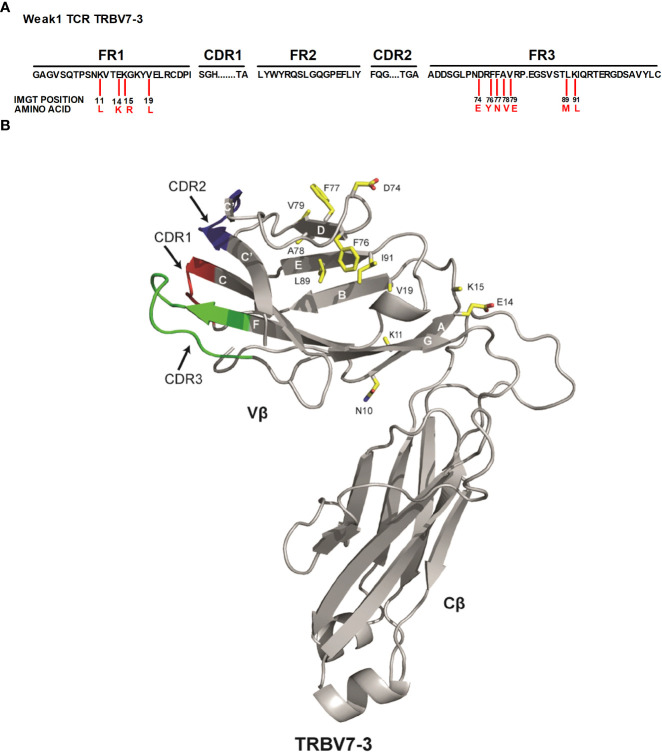
Identified 11 TRBV framework residues are distal to the CDR parts **(A)** Representation of the candidate residues assessed. Numbers in black indicate the IMGT positions in the TCRβ framework regions that were substituted with the amino acids indicated in red. FR framework region, CDR complementarity determining region. **(B)** The published 3-D structure of the 3PL6 TCR (TRAV13-1/TRBV7-3) was used as a model for the weak 1 TCR (TRAV13-2/TRBV7-3) used in this study. The location of each of the 11 residues that were changed in the weak 1 TCR Vβ domain to enhance TCR surface expression is indicated. Also included is location of residue at position 10.

In order to select additional candidate residues in the TRBV framework, we exploited a previous bioinformatics analysis that identified amino acid positions that were highly enriched in a library of more than 130,000 TCRs with a ‘dominant’ expression profile compared to a similar number of TCRs with a ‘weak’ expression profile (16). We had previously employed this analysis to identify framework mutations at TCR-α96, TCR-β9, and TCR-β10 that substantially increased TCR expression and functionality (16). Here, we focussed on 11 additional amino acid residues in TCR-β that showed a highly significant enrichment in the ‘dominant’ library, were distal to the CDR1, 2 and 3 loops (**Figures 1A, B**), and based on TCR structural modelling were predicted to affect the stability of the Vβ domain. We mutated a TCR from the ‘weak’ expression library comprised of TRAV13-2 and TRBV7-3 (weak 1-TCR), changing the amino acids of the selected candidate residues to those present in the ‘dominant’ library.

**Figure 2:**
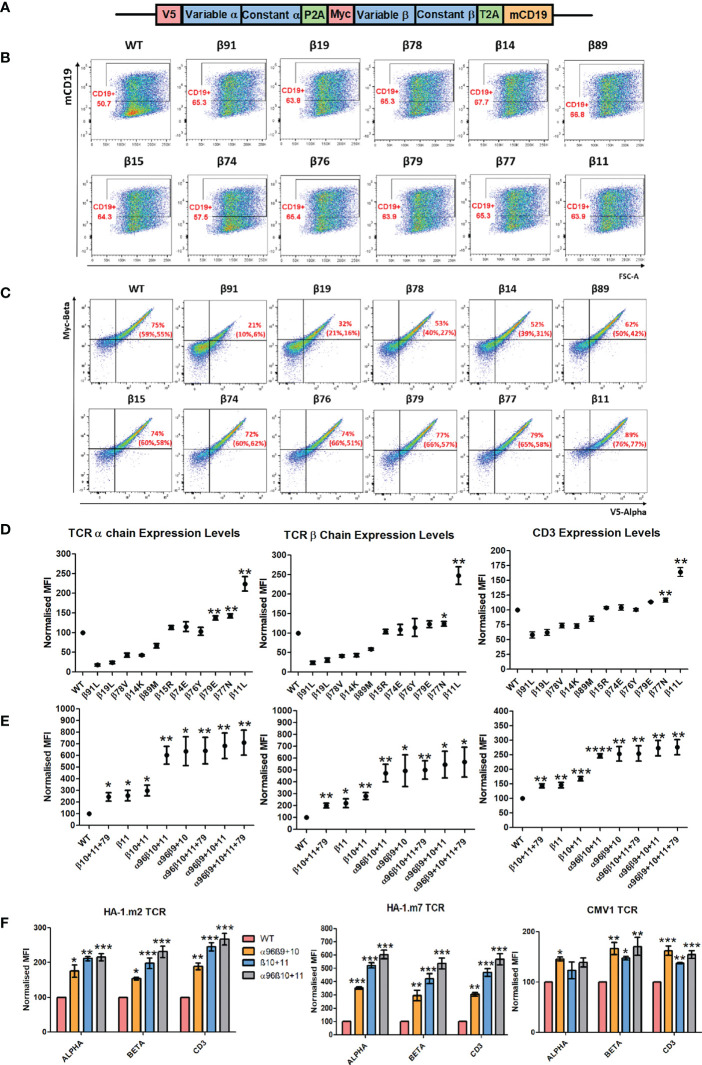
Single and combinations of amino-acid TRBV framework residue replacements can improve TCR expression in Jurkat76 cells. **(A)** Schematic representation of the retroviral vector that was used to transfer TCRs into Jurkat76 and primary T cells. Anti-V5 and anti-Myc Abs were used to determine the expression levels of TCRα and TCRβ respectively. Anti-murine CD19 Abs were used to determine transduction efficiency. mCD19 truncated murine CD19 sequence. **(B)** Representative example of three independent experiments showing Jurkat76 cells transduced with either the weak1 wild-type (WT) TCR or weak TCR constructs with the indicated single TCRβ chain amino-acid residue swap. Shown is mCD19 expression levels, indicating transduction efficiency. **(C)** Representative plots of three independent experiments showing TCRα and TCRβ expression in CD19+ gated Jurkat76 cells expressing WT or single amino-acid TCRβ chain residue modified versions of the Weak1 TCR. Numbers in brackets demonstrate the percentage of introduced TCR+ cells of the repeated experiments **(D)** Representative graphs of three independent experiments showing MFI (median fluorescence intensity) of TCRα, TCRβ and CD3 expression in CD19+ gated Jurkat76 cells transduced with WT or single amino-acid TCRβ versions of the weak1 TCR. MFI (Mean+/- SEM) data has been normalised to WT expression. Unpaired t test was applied, *: p<0.05, **: p<0.01 **(E)** Representative graphs of three independent experiments showing MFI of TCRα, TCRβ and CD3 expression in CD19+ gated Jurkat76 cells transduced with either weak1 WT TCR or TCRs with either 1 amino-acid TCRβ chain residue change, or residue combinations as indicated. MFI (Mean+/- SEM) data has been normalised to the WT TCR expression. Unpaired t test was applied, *: p<0.05, **: p<0.01 **(F)** Jurkat76 cells were transduced with either WT TCRs or 3 TCR versions with the indicated residue modifications for 3 different antigen specific TCRs (HA1.m2, HA1.m7 and CMV1 pp65). Shown is a representative example of three independent experiments showing MFI values of TCRα, TCRβ and CD3 in CD19+ gated cells. MFI (Mean+/- SEM) data has been normalised to WT expression. One-way ANOVA, Dunnett’s Multiple Comparison Test, *: p<0.05, **: p<0.01, ***: p<0.001 (Residue changes were as followed: HA1.m2: α96P>L, β10H>Y, β11K>L, HA1.m7: α96T>L, β10H>Y, β11K>L.

*In vitro* mutagenesis was employed to substitute these residues in the weak TCR, followed by transduction into human Jurkat76 cells to assess TCR expression levels. Truncated murineCD19 (mCD19) was used to identify transduced cells, and V5 and Myc tags located at the N-terminus of the TCRα and TCRβ chain, respectively, were used to measure the expression levels of each TCR chain (**Figures 2A–C**). One of the candidate residues tested (mutant β11) was able to significantly increase TCR expression levels in Jurkat76 cells (**Figures 2C, D**). The single amino acid change from lysine to leucine at position Vβ-11 resulted in a 2-fold increase in TCRβ, TCRα and CD3 expression levels in Jurkat76 cells (**Figure 2D**).

### Combinations of amino acid changes can further improve TCR expression in Jurkat76 cells

Next, we tested whether combinations of amino acid changes could further improve expression of the weak TCR. Combining the change of Vβ residue 11 with the previously identified TCRβ10 (N>Y) mutation that is predicted to enhance the stability of Vβ-Cβ interaction (16), resulted in a small, but non-significant improvement of TCR expression compared to the single amino acid change at position 11 only (**Figure 2E**). However, combining various Vβ modifications with the TCR-α96 framework mutation previously identified (a single amino acid change from proline (P) to leucine (L) at position 96 of the Vα domain) doubled TCRα, β and CD3 expression levels compared to modifications in the Vβ domain alone. In the weak1 TCR, all tested Vβ modifications combined with L96 in the Vα domain achieved similar high levels of expression in Jurkat76 cells.

Next, we tested which modifications are best able to achieve optimal expression of three HLA-A0201-restricted TCRs specific for the minor histocompatibility antigen HA-1 or for cytomegalovirus (CMV). All TCRs demonstrated improved expression when only Vβ residues 10 and 11 were modified, with the most impressive improvement seen with the HA-1.m7 TCR, followed by the HA1.m2 and CMV1 TCR. Adding the TCR-α96 (P>L) modification to the TCR-β10-11 mutated chains of the 3 antigen-specific TCRs only marginally improved the expression levels (**Figure 2F**). This indicates that the impact of introducing leucine 96 in Vα is TCR dependent, as it increased expression of the weak1-TCR substantially (**Figure 2E**), but only marginally increasing the expression of the three antigen-specific TCRs (**Figure 2F**). Of note, the modifications had a relative small effect on the CMV TCR, enhancing expression by only 1.5-fold, while the same modifications enhanced HA-1.m7 TCR expression by 6-fold (**Figure 2F**). This is probably due to the fact that the unmodified CMV TCR already displays strong surface expression, while the HA-1.m7 TCR is poorly expressed in the absence of Vα and Vβ modifications.

**Figure 3:**
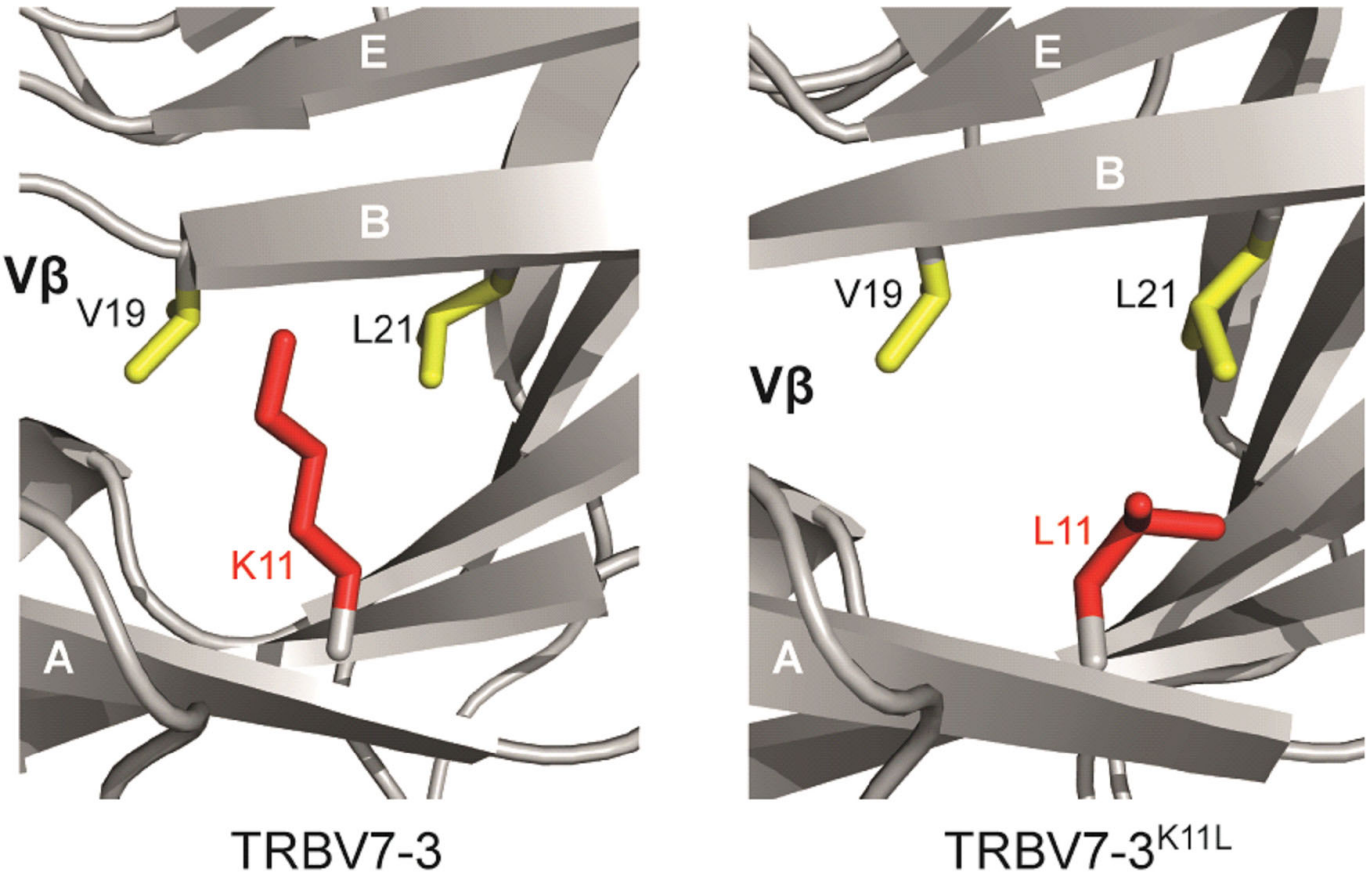
Structural modelling provides insight into the mechanistic role of framework residues in TCR stability. The change of K11β to L11β improves non-polar interactions within a hydrophobic core of the β chain. Left hand figure, lysine at position 11. Right hand figure, leucine at position 11.

Our modelling approaches highlighted a likely molecular mechanism underlying this effect (**Figure 3**). K11β is a semi-buried residue that protrudes from strand A and its positively charged side chain is in close proximity to the non-polar Vβ domain core region. The positive effect of L11β on TCR expression can be explained by its protrusion from strand A into the hydrophobic core. Replacing K11β with L11β predicts that the leucine side chain is likely to stabilise the hydrophobic core by mediating multiple non-polar interactions with V19β (strand B) and L23β (strand B) (**Figure 3**). Therefore, the L11β substitution likely enhances the stabilisation of the hydrophobic core of the Vβ domain.

### TCR modifications improve expression and reduce mis-pairing in primary human T cells

**Figure 4:**
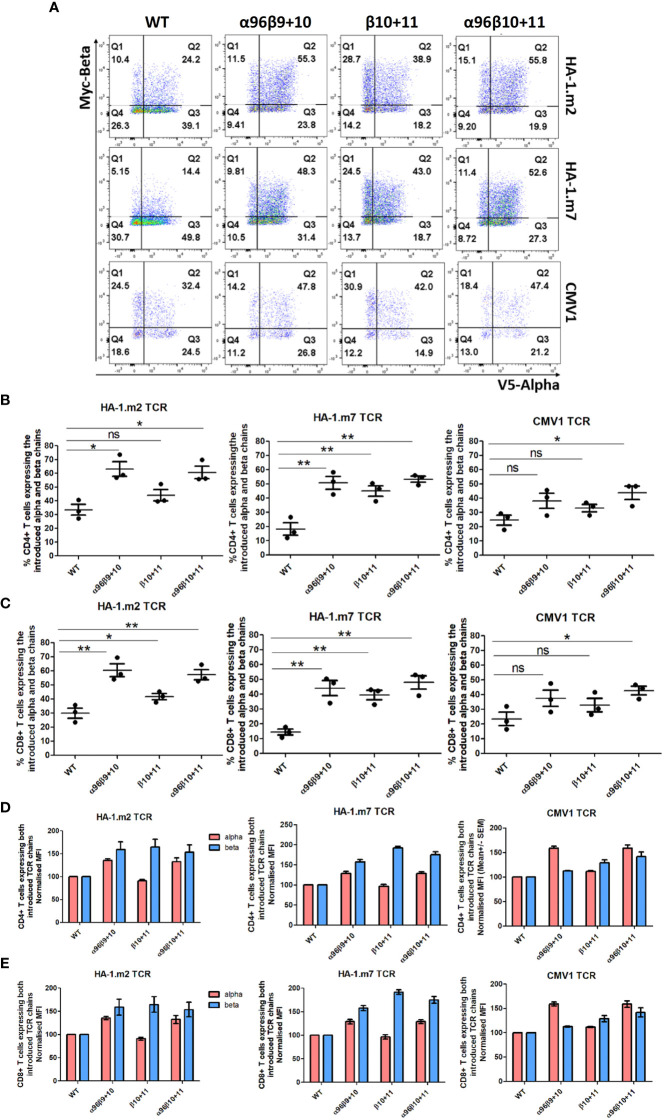
Alpha and beta chain residue modifications elevated introduced TCRs expression and reduced mis-pairing in human primary T cells. Human activated PBMCs were transduced with either WT TCR or TCRs with the indicated modified residue changes for 3 antigen specific TCRs (CMV1 pp65, HA1.2 and HA1.m7). Shown are representative examples of three independent experiments. **(A)** FACS plot show introduced TCRα chain and TCRβ chain expression in CD8+CD19+ gated cells. **(B)** Graphs show the percentage of cells expressing both the introduced α chain and introduced β chain in CD4+CD19+ gated T cells. Unpaired t test, *: p<0.05, **: p<0.01, ns: non-significant. **(C)** Graph show the percentage of cells expressing both the introduced α chain and introduced β chain in CD8-CD19+ gated T cells. Unpaired t test, *: p<0.05, **: p<0.01, ns: non-significant **(D)** The MFI (Mean+/- SEM) values of the introduced TCRα and TCRβ chain in CD8+CD19+ gated T cells. MFI values are normalised to WT expression. **(E)** The MFI (Mean+/- SEM) values of the introduced TCRα and TCRβ chain in CD4+CD19+ gated T cells. MFI values are normalised to WT expression.

In the next set of experiments, we assessed how modifications of the three antigen-specific TCRs above affected their expression and mis-pairing in primary human T cells (**Figure 4A**). T cells were transduced with wild type TCRs or with versions containing Vβ modifications, either alone or in combination with the TCR-α96 (P>L) Vα domain modification. Following flow cytometry, transduced cells were identified by gating on CD19+ T cells, and levels of V5 and myc staining served to assess expression of the introduced α and β chain, respectively. The analyses demonstrated that transduction of wild type TCRs generated T cells that mostly expressed mis-paired TCRs consisting of introduced α and endogenous β chain (**Figure 4A**, Q3 in the FACS plots), or introduced β and endogenous α chain (Q1). All modifications increased the number of T cells expressing both the introduced α as well as the introduced β chain (Q2). Although the modification of position 10 and 11 of Vβ increased the number of T cells in Q2 expressing the introduced α and β chains, it also increased mis-pairing between the modified β and the endogenous α chain (Q1). For all three TCRs tested the modification of both Vβ and Vα was required to increase the number of T cells expressing both chains, and also reduce TCR mis-pairing (**Figure 4A**). **Figures 4B, C** display the summary of TCR expression in gated CD4+ T cells and in CD8+ T cells, respectively. It shows that the previously identified changes at 96α,9β,10β and the new combination of 96α, 10β, 11β were equally effective in increasing the percentage of T cells expressing both introduced TCR chains, except for the CMV1 TCR where only the new combination of 96α, 10β, 11β significantly increased CD4+ and CD8+ T cell numbers expressing both TCR chains. Finally, **Figures 4D, E** illustrates that the TCR modifications not only increased the numbers of CD4+ and CD8+ T cells expressing both chains, but the displayed MFI values indicate that the surface expression levels of the introduced chains was also increased compared to the MFI seen with the wild type TCRs. Together, the data show that the TCR modifications increased both the number of T cells expressing both chains, as well as the amount of TCR found on the surface of these T cells.

### TCR modifications improve antigen-specific effector function

**Figure 5:**
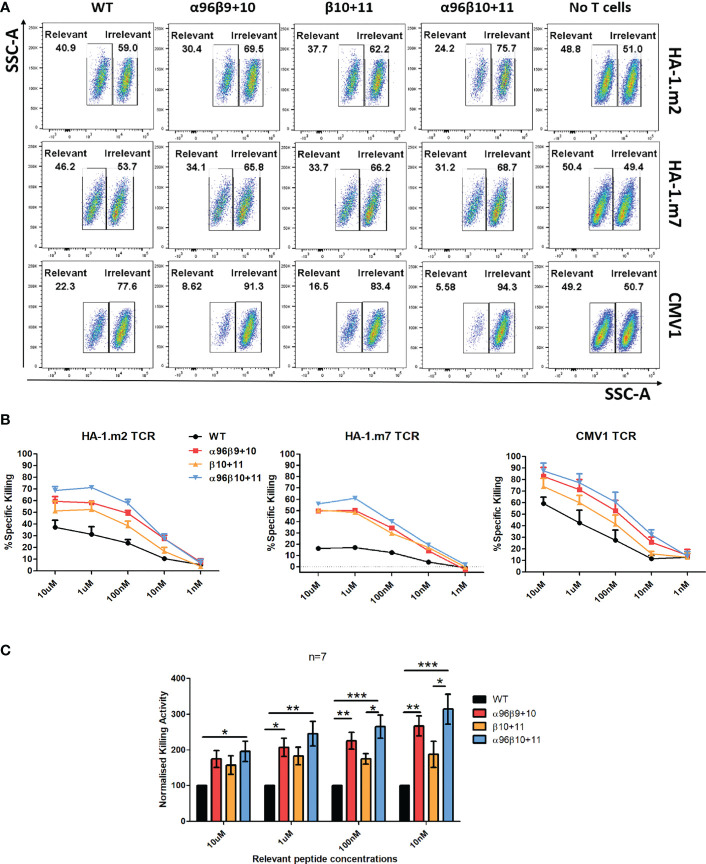
Enhanced cytotoxicity was observed with T cells expressing the residue modified TCRs. Human activated PBMCs were transduced and rested for 8-10 days and then used in subsequent assays. **(A)** Cell were co-cultured with T2 cells labelled with 10μM cognate peptide (low CFSE) or control peptide (high CFSE) mixed at 1:1 ratio (Effector: Target = 2:1). **(B)** Cell were co-cultured T2 cells labelled with 10μM – 1nM cognate peptide (low CFSE) or control peptide (high CFSE) mixed at 1:1 ratio (Effector: Target = 2:1). The following day, cells were stained for CD3 and Live/Dead and acquisition was collected on FACS. ‘No T cells’ control is 1:1 cognate or control peptide pulsed, CFSE labelled T2 cells only. Representative graphs demonstrating the specific killing activity of T cells transduced with either wild type or residue modified versions of HA-1.m2 (n=3), HA-1.m7 (n=1) and CMV1 (n=3) TCRs. Specific killing is: %= {100- [(Relevant/Irrelevant with T cells)/(Relevant/Irrelevant with no T cells)]*100} **(C)** Pooled relative killing data of all three TCRs tested in 7 independent experiments. At each peptide concentration the killing activity of the modified TCRs is relative to the killing activity seen with each wild type TCR which is set as 100. One-way ANOVA, Tukey Multiple Comparison Test was applied, *: p<0.05, **: p<0.01, ***: p<0.001.

In the final set of experiments, we tested whether TCR modification improved the antigen-specific killing activity of primary human T cells. Transduced T cells were co-cultured with CFSE-high target cells pulsed with an irrelevant peptide, and CFSE-low targets pulsed with the TCR-recognised cognate peptide. Although T cells transduced with wild type TCRs were able to kill the relevant target cells, the modified TCRs displayed much improved killing activity (**Figure 5A**). Vβ modification alone resulted in improved killing, but the most efficient antigen-specific killing was seen when the Vβ and the Vα96 modifications were combined (**Figures 5A, B**). Interestingly, in all experiments the TCRs modified at the positions 96α,10β,11β showed slightly higher killing activities compared with the previously identified combination 96α,9β,10β. A comparison of the pooled killing data of all three TCRs showed that the 96α,10β,11β modification identified in this study displayed the most significant improvement in target cell killing at all peptide concentrations tested (**Figure 5C**).

## Discussion

In this study, we have demonstrated that modifying several framework residues away from CDR1, 2 and 3 can improve TCR expression and T cell antigen specific function, while at the same time reducing mis-pairing of the introduced and endogenous TCR chains. We selected 11 candidate Vβ residues by analysing our previously created bioinformatic dataset and candidate TCR structure. TCR-deficient Jurkat76 cells and primary human T cells were transduced with modified TCRs to identify the effects of each residue change on TCR expression. Results indicated that a single amino acid change at the 11^th^ position of the TCRVβ domain resulted in a 2-fold increase of the TCR and CD3 in Jurkat76 cells. Combination with several other Vβ residue changes did not lead to any significant improvement compared to Vβ 11 only. However, introduction of the Vα 96 P>L modification we identified previously along with several β chain modifications enhanced expression of the Weak1 TCR.

We know from our previous work (16) that framework amino acid modifications do not cause any alteration in the mRNA expression levels of the introduced TCRs. It is well established that following their production, TCRα and TCRβ chains complete their pairing in the ER. Interaction of these two paired TCR chains with CD3 is pivotal to maintain their intact structure, otherwise they are degraded (40). Jurkat76 cell experiments indicated that not all TCRα and TCRβ chains produced in the ER migrate to the cell surface. Even though the TCR chains are produced in the ER, some may not complete proper folding to become fully functional TCR proteins; alternatively, they may complete folding, but because of low stability, they may not pair efficiently and subsequently undergo degradation in the ER. It is likely that residue substitutions enhancing TCR surface expression play a role in improving the folding and stability of the nascent TCR chains, thereby facilitating heterodimeric pairing and assembly with CD3 chains, ultimately enhancing migration of the residue modified TCRs to the T cell surface. Surprisingly, a number of candidate TCRβ framework mutations we tested caused a reduction in TCR expression, despite the fact they were identified as enriched in ‘strong’ TCRs, and appeared to be structurally relatively conservative. One possible explanation is that dominant TCR libraries show enrichment of complete V gene sequences that contain residues that drive high TCR folding, stability and expression, but may also contain genetically linked V gene residues that may impair TCR expression. Consistent with this possible explanation, we previously demonstrated that some amino acid residues enriched in dominant TCR-Vα chains did impair TCR surface expression, and that changing these residues to amino acids that were present in weak TCRs did actually improve TCR expression (16).

Primary human T cell experiments with HA-1.m2, HA-1.m7 and CMV1 TCRs revealed that the same residue changes elicited similar improvements in CD4+ and CD8+ T cell numbers bearing the introduced TCRs and their expression levels. By assessing the presence of V5 and Myc tags at the N-terminus site of TCRα and TCRβ, respectively, the expression level of each TCR chain could be measured independently. That also enabled assessment of cell numbers expressing mis-paired TCRs. All residue modified constructs increased the number of T cells expressing both introduced TCR chains compared to that of wild type TCRs. In addition, residue modifications decreased the number of cells expressing the mis-paired TCRs, while the wild type form of each antigen specific TCR displayed higher number of cells expressing either introduced-β-endogenous-α or vice versa mis-paired versions. We observed that modifications of both TCRα and TCRβ are required to improve the expression of the introduced TCR chains while ensuring less mis-paired TCR formations. Published work has shown that codon optimisation and the replacement of the human TCR α/β constant domains with murine domains can reduce mis-pairing and increase expression of correctly paired TCRs. In pilot experiments we saw that the TCR framework engineering approach described here improves correct TCR pairing more effectively than codon optimisation. Although murine constant regions were most effective in improving correct TCR pairing in human T cells, this approach is not suitable for clinical application as murine constant domains are immunogenic and likely to cause rejection of the engineered T cells in patients. To reduce the rejection risk, groups have identified a minimal set of 9 murine residues that were sufficient to enhance TCR expression in human T cells (41, 42). Our preliminary data showed that TCRs containing 3 residue changes in the variable framework region were more efficiently expressed in human T cells than TCRs containing the minimal set of 9 murine residues in the constant region. This suggests that the framework technology described here is superior to the previously described ‘murinization’ technology in terms of TCR expression and reduction of immunogenicity related to 3 amino acid changes compared to 9 residue alterations. Another important observation with residue modifications was that they conferred increased dominance to the TCRs. Transduction of TCRs into polyclonal primary human T cells provided a means to assess dominance, given the T cell repertoire naturally contains an immense variety of different TCRs, with diverse expression profiles ranging from weak to strong. These experiments clearly indicated that relative to wild type TCRs, introduction of residue modified TCRs decreased the percentage of cells expressing solely naturally dominant endogenous TCRs. Therefore, residue modification conferred increased dominance to the introduced TCRs, allowing more successful competition with the endogenous TCR repertoire, and ultimately increasing percentage expression.

We have also observed that the impact of residue modifications on TCR expression and T cell function may vary depending on the initial quality of a TCR. While the performance of TCRs with a ‘weak’ expression profile can be elevated dramatically by residue modification, the effects may be more limited on TCRs with a ‘strong’ expression profile. We recorded remarkably low HA-1.m7 wild type TCR expression in TCR-deficient Jurkat76 cells, suggesting that even in the absence of an endogenous competitor, this TCR did not form a TCR complex efficiently. Nevertheless, with framework residue modifications (α96β10+11), its performance was substantially improved in both Jurkat76 cells and primary T cells, resulting in increased numbers of T cells expressing the introduced TCR, 10-fold increased antigen-specific cytokine production, and augmented cytolysis. While the CMV1 TCR, which is a strong TCR based on Jurkat76 cell experiments and wild type TCR functional assay results, also benefited from the residue changes (with α96β10+11) in all the categories, performance gains were limited relative to the modified HA-1.m7 TCR.

Another advantage of residue modification is that it endows T cells with increased sensitivity. Intracellular cytokine staining demonstrated that modified TCRs retained peptide specific cytokine production without non-specific activity against irrelevant peptide (**Supplementary Figure 1**). In killing assays, we observed an increased sensitivity of T cells expressing the residue modified TCRs. Killing assay results indicated more than 100-fold increase in antigen sensitivity for T cells bearing residue modified (α96β10+11) HA-1 TCRs. This probably arises from the enhancements observed in the TCR expression level of the introduced TCRs. As the density of the antigen specific TCRs on the T cell surface increases, decreased antigen becomes sufficient to elicit an antigen specific response. Considering the hostile tumour environment in which there may be a scarcity of tumour specific or tumour associated antigen presentation to T cells, framework engineering seems promising route to equip T cells with an increased target sensitivity.

In this study, we have demonstrated that by substituting as few as three amino acids in the framework region of TCR variable domains, it is possible to improve the expression level of the introduced TCR and ultimately augment T cell antigen specific function. We observed that TCRα and TCRβ framework residue modifications are required for an optimal TCR expression and enhanced T cell function. The ultimate goal of TCR-T therapy relies on achieving expression of antigen specific TCR in T cells as effectively and safely as possible. Integration of framework engineering technology into this therapeutic approach holds substantial promise, namely to further exploit the potential of TCR therapy by augmenting both its efficacy and safety.

## Data availability statement

The raw data supporting the conclusions of this article will be made available by the authors, without undue reservation.

## Author contributions

AD designed and conducted experiments, analysed data and wrote the paper. ST designed experiments and wrote the paper. FM and BW designed experiments and wrote the paper. HS initiated the study, designed experiments, analysed data and wrote the paper. All authors contributed to the article and approved the submitted version.
