# Supplementary material for: Modifications outside CDR1, 2 and 3 of the TCR variable β domain increase TCR expression and antigen-specific function
Source: Front Immunol. 2023 Apr 12;14:1148890. doi: 10.3389/fimmu.2023.1148890 (PMC10134071; doi:10.3389/fimmu.2023.1148890)
Supplement: Supplementary file 1 [file DataSheet_1.pdf]

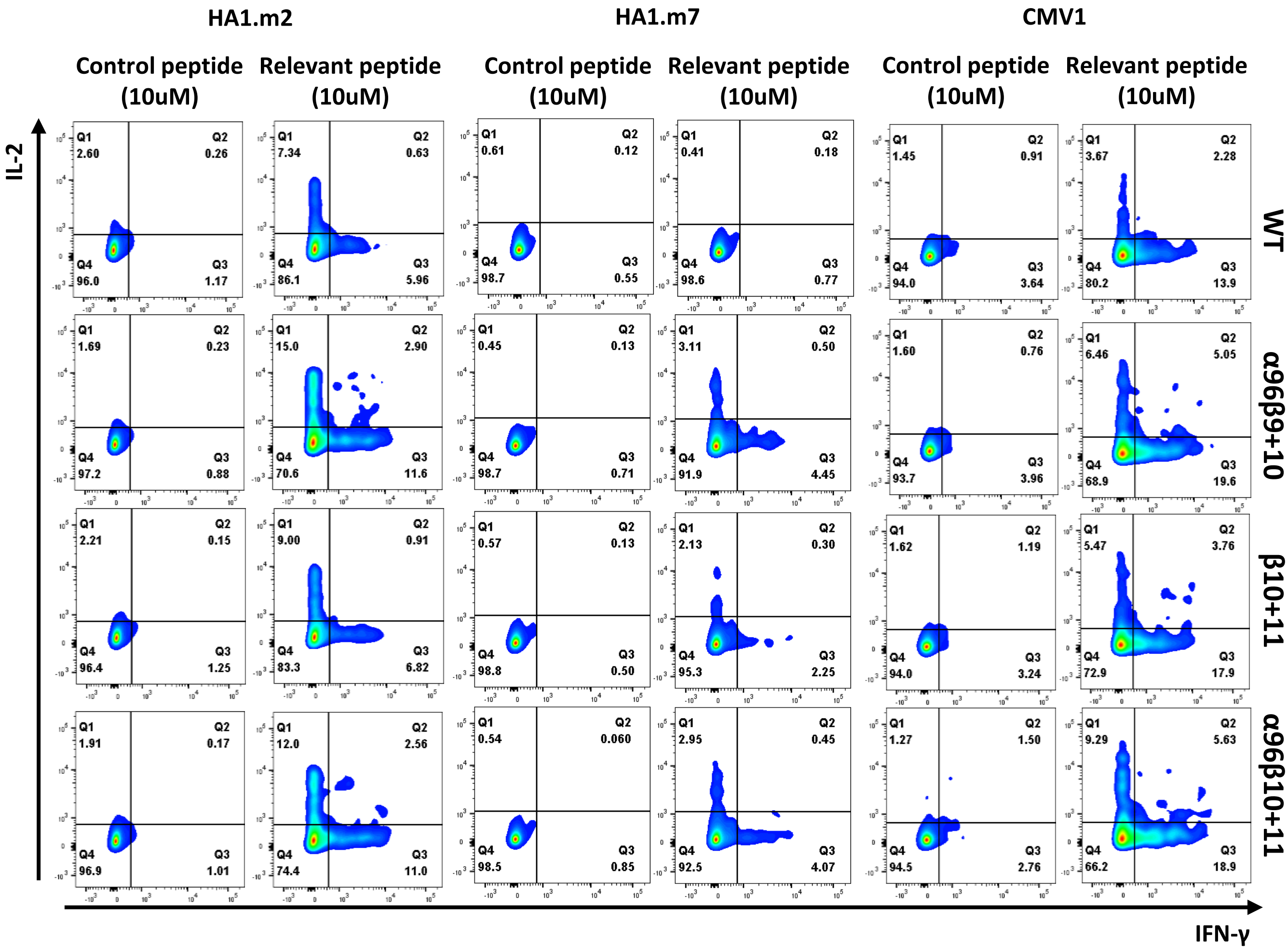

Supplementary Figure 1. Modified TCRs retained peptide specificity. Human activated PBMCs were transduced with the indicated TCRs and rested for 8-10 days and then stimulated with T2 cells pulse with the TCR-recognised peptide or with control peptide (10uM each). After 18h stimulation intracellular cytokine staining was performed and flow cytometry was used to demonstrate peptide-specific IL-2 and IFN-γ production by wild-type TCRs and by modified TCRs (n=1).
